# CRISPRs provide broad and robust protection to oral microbial flora of gingival health against bacteriophage challenge

**DOI:** 10.1007/s13238-015-0182-0

**Published:** 2015-06-30

**Authors:** Huiyue Zhou, Hui Zhao, Jiayong Zheng, Yuan Gao, Yanming Zhang, Fangqing Zhao, Jinfeng Wang

**Affiliations:** Computational Genomics Lab, Beijing Institutes of Life Science, Chinese Academy of Sciences, Beijing, 100101 China; University of Chinese Academy of Sciences, Beijing, 100049 China; Wenzhou People’s Hospital, Wenzhou, 325000 China

**Dear Editor,**

Clustered regularly interspaced short palindromic repeats (CRISPR), which are widely present in prokaryotic genomes (Grissa et al., 2007), belong to a family of DNA sequences characterized as short direct repeats (DR) separated by spacers (Jansen et al., 2002). CRISPR and CRISPR-associated (cas) genes are involved in resistance against exogenous sequences, and recognition of infected bacteriophages depends on the sequence similarity between spacers and targeted phage DNA segments (Barrangou et al., 2007). CRISPRs exhibited diversity in different microbial communities, and that may be the response to different phage infections (Tyson and Banfield, 2008). Dynamic change of spacers in CRIPSR reflects altered resistance to viruses (Andersson and Banfield, 2008), which may play important roles in shaping the structure and driving the evolution of microbial communities. Consequently, in addition to a tool designed for genome editing (Ghorbal et al., 2014), CRISPRs are widely used to demonstrate the dynamic changes of bacteria and phage communities.

In human microbial communities, a study involving more than 700 datasets of metagenomic sequencing reported that only a few spacers were shared among different body sites or individuals although known CRISPRs can be found in most human oral microbiome (Rho et al., 2012). A study on oral microbiome also found that less than 2% spacers were shared between healthy people as a result of different people may encounter different virus population (Pride et al., 2011). Comparison between oral CRISPR sequences and virome further indicated that viruses and CRISPR diverged significantly among individuals and a large proportion of spacers were specific to each individual and time point (Pride et al., 2012). Another study on CRISPR suggested that spacers from oral bacteria were associated with oral viral ecology (Robles-Sikisaka et al., 2013). These results reveal that CRISPRs were under pressure of dynamic change of viruses in oral environment. Despite the potential effect on oral microbial ecology, little attention was paid to the comparison between CRISPRs under disease and health status until now. As oral cavity is a very complicated environment with diverse bacterial communities and periodontitis is a common disease closely related to microbial flora disorders (Belda-Ferre et al., 2012), we focused on the comparison of CRISPRs of bacterial communities under different periodontal status (in periodontal health and in periodontitis) in this study.

To characterize the CRISPR compositions under different periodontal status and the relationship between healthy and periodontitis patients, we recruited 9 human subjects suffered from periodontitis and 9 health controls. They were selected from mature non-smoking females among 30~60 years old without any other systemic disease. Periodontal status of these volunteers was clinical monitored at six sites per tooth by a periodontist. Probing depth and attachment loss were taken as the main classification criteria. Chronic periodontitis was selected whose periodontal pockets ≥4 mm and attachment loss ≥6 mm at more than 4 tooth sites. Periodontal health had no probing depth >2 mm or attachment loss >2 mm at any site. Dental plaques were individually collected at least 2 h after eating and 6 h after tooth brushing, and DNA was extracted and then was disrupted into fragments with ~180 bp in length. To assess the composition of the microbial communities, they were sequenced to 2× 100 bp paired-end (PE) reads by an Illumina HiSeq 2000 sequencing instrument. In total, we got 176,931,096 reads for 18 samples (Table S1). Previous studies always assemble the reads to contigs to identify CRIPSR arrays, but part of the reads will be omitted in the assembly. To fulfill all the information of the reads, we identified DRs and spacers directly from raw reads by Crass 0.3.12 (Skennerton et al., 2013) which is on the basis of the distinctive structure of CRISPR. By this step we got 844 DR and 24,841 spacer sequences. The length of DRs and spacers mostly distributed from 30 bp to 40 bp (Fig. 1A). To classify these CRISPR elements, DRs and spacers were respectively aligned to bacteria and phage genomes in NCBI non-redundant (NR) database.

**Figure 1:**
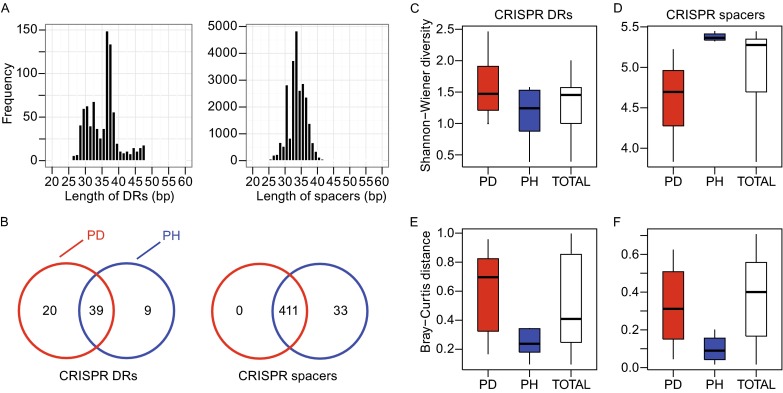
**The composition of the CRISPRs in samples collected from patients with chronic periodontitis (PD) and people with healthy gingiva (PH).** (A) The distribution of the length of DRs (left panel) and spacers (right panel) identified from metagenomic sequencing data. (B) Numbers of DRs (left panel) and spacers (right panel) in PD (red circle) and PH (blue circle). (C) Shannon-Wiener diversities of the DRs in PD (red box), PH (blue box) and all (white box). (D) Shannon-Wiener diversities of the spacers. (E) Bray-Curtis diversities of the DRs. (F) Bray-Curtis diversities of the spacers. The boxes represent the interquartile range between the first and third quartiles. The whiskers denote the lowest and highest values within the interquartile ranges of the first and third quartiles. The thick lines inside the boxes represent the medians.

Accordingly, DRs were assigned to 68 bacterial genera (Table S2), with 48 in periodontal health (PH) and 59 in periodontal disease (PD). Spacers were assigned to 444 phage species (444 in PH and 411 in PD, Table S3). Though more DRs were found in PD, PH possesses 33 more spacers than PD (Fig. 1B). Moreover, PH had more spacer-contained reads in 386 of 444 phage species. This result suggests that microbiome from healthy people had a wider spectrum in resisting the invasion of phages than patients suffered from periodontitis. When the oral microbial flora of patients suffered from chronic periodontitis encountered bacteriophages corresponding to the distinctive spacers hold only by healthy people, it is easier to be attacked and might not maintain a stable bacterial community, and microbiota disequilibrium was exactly the reason causing or increasing the susceptibility of periodontal diseases (Curtis et al., 2011). As expected, DRs in PD showed slightly higher Shannon-Wiener diversities than in PH (Fig. 1C) and the spacer composition exhibit significantly higher diversities in PH (Mann-Whitney U test *P*-value = 0.0002, Fig. 1D) on the contrary. For Bray-Curtis distance, both DRs (*P*-value = 0.0001) and spacers (*P*-value < 0.0001) have remarkably higher values in patients (Fig. 1E and 1F). Principle components analysis (PCA) also indicated CRISPR composition distinguished the periodontal status of the oral cavities (Figs. S1 and S2). PH samples had similar composition of DRs and spacers, and thus PH may have more stable microbial CRIPSRs with a large variety of spacers which can defend the invasion from bacteriophages and subsequently preserve the dynamic balance of the whole microbial community.

To characterize the subjects that play a role of defense, we ranked the DRs based on the relative abundance of DRs-assigned genera. The relative abundance of each genus was calculated by dividing total reads numbers of each sample and then normalized to 100,000. Top 30 most abundant genera of DR source were shown in the bubble chart (Fig. 2A), which accounted for 98.8% of the entire DRs. Common bacteria of human oral cavities such as *Corynebacterium*, *Actinobacillus*, *Capnocytophaga*, *Streptococcus* and *Prevotella*, were all included in this consortia. However, *Streptococcus* and *Prevotella*, which usually account for the dominant of oral microbiota, are not the leading contributors of DRs. This result suggests the abundance of CRISPRs of microbial community in human oral cavities is not only determined by the amount of bacteria, but also closely related with species composition. Previous studies have reported that some bacteria were highly abundant in periodontitis, such as *Prevotella*, *Selenomonas* and *Treponema* (Wang et al., 2013). *Porphyromonas gingivalis*, *Tannerella forsythia* and *Treponema denticola* were also widely known as ‘the red complex’ to be involved in the periodontal diseases (Darveau, 2010). Although we found that the DR abundance of several genera (e.g., *Prevotella*, *Selenomonas*, *Treponema* and *Tannerella*) in PD samples was a little higher than that in PH samples, we did not observe any of them with significant difference by Mann-Whitney U test. This reveals that the ability of defense to phages is at least not weaker in healthy people than in patients.

**Figure 2:**
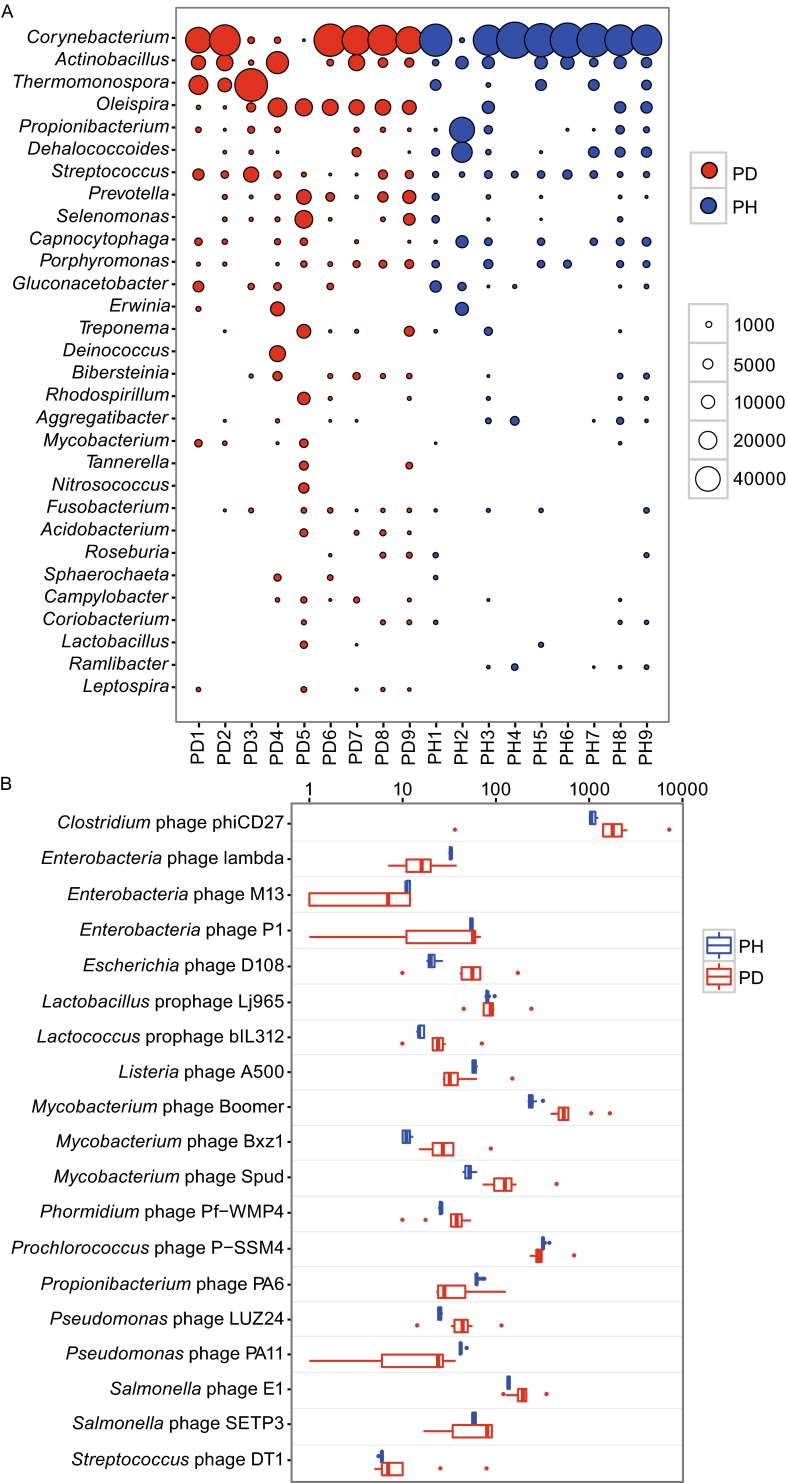
**Evenness of the CRISPR DRs and spacers in PD and PH samples.** (A) Top 30 most abundant bacterial genera of DR source. Area of each bubble represented the relative abundance (by dividing total reads numbers of each sample and then normalized to 100,000) of DRs for each sample (column) and each genus (row). Red color represented PD (*n* = 9) and blue color represented the PH (*n* = 9) samples. (B) The most variegated spacers (classified to phage species by BLASTX) between the PD and PH groups (*n* = 9). Scales on x-axis represent relative abundance.

To further investigate the primary cause of significant differences in CRISPR elements between periodontally diseased patients and healthy people, we conducted F-test to compare the abundance variation of each DRs and spacers. We used 0.0007 (0.05/68) and 0.001 (0.05/32) as *P*-value cutoffs to perform the Bonferroni correction for multiple testing of DRs (68 genera) and spacers (32 genera), respectively. For 68 bacterial genera carrying DRs, abundance variation of 32 genera was significantly lower in health people than in patients (Fig. S3). We also observed a similar situation for spacers (Fig. 2B). The spacers aligned to 305 of 444 phage species had greater concordance in healthy people, and they occupied 69.2% of total spacers. 96 species were left even with *P*-value cutoff 0.0001. The most substantial variation came from the species *Clostridium* phage phiCD27. We also classified these spacers to the genus level and got a similar result. 23 out of 28 genera had greater concordance in healthy people, and they occupied 91.8% of all the spacers. Since spacers indicate the ability of bacteria to resist invasion of certain bacteriophages, and thus the microbiome in healthy people seems to be fairly adapted to defend invasion of such phages and could be much more robust to micro-environment alteration.

In conclusion, by systematical analysis of CRISPR using whole genome sequencing data for oral microbiome, we found the composition of DRs and spacers are significantly different between PD and PH. The defense potentials by CRISPRs were related to both evenness and richness of oral bacteria. Healthy people had less DRs and more spacers and were more similar with each other, which will assemble a robust and functional bacterial community to resist the invasion from phage. CRISPRs may play some roles in the equilibrium of microbial community. However, the details of how they function in the oral ecology still need more exploration.

## FOOTNOTES

This work was supported by the National Natural Science Foundation of China (Grant No. 31300110) to J. Wang and (Grant No. 31301031) to Y. Zhang. We thank L. Hou and L. Zhang (Research Facility Center at Beijing Institutes of Life Science, Chinese Academy of Science) for their sequencing assistance.

Huiyue Zhou, Hui Zhao, Jiayong Zheng, Yuan Gao, Yanming Zhang, Fangqing Zhao and Jinfeng Wang declare that they have no conflict of interest. All procedures followed were in accordance with the ethical standards of the responsible committee on human experimentation (Beijing Institutes of Life Science, CAS) and with the Helsinki Declaration of 1975, as revised in 2000 (5). Informed consent was obtained from all patients for being included in the study.

## Electronic supplementary material

Supplementary material 1 (DOCX 15 kb)

Supplementary material 2 (XLSX 14 kb)

Supplementary material 3 (XLSX 56 kb)

Supplementary Figure S1 Principle components analysis (PCA) based on the DRs using imDEV. Each *red dot* represented one PD sample (*n* = 9) and each *blue dot* represented one PH sample (*n* = 9). *Grey dots* represented DRs taxa (68 genera in total) used for PCA. *Discrete dots* indicated the DRs that can better separate the samples (PSD 582 kb)

Supplementary Figure S2 Principle components analysis (PCA) based on the spacers. Each *red dot* represented one PD sample (*n* = 9) and each *blue dot* represented one PH sample (*n* = 9). *Grey dots* represented assigned bacteriophages (414 in total). *Discrete dots* indicated the bacteriophages that can better separate the samples (PSD 603 KB)

Supplementary Figure S3 The most variegated DRs (classified to bacterial genera by BLASTX) between the PD and PH samples. *Red color* represented PD (*n* = 9) and *blue color* represented PH (*n* = 9) samples. Scales on x-axis represent relative abundance (PSD 529 KB)
