# Supplementary material for: CRISPRs provide broad and robust protection to oral microbial flora of gingival health against bacteriophage challenge
Source: Protein Cell. 2015 Jun 30;6(7):541–5. doi: 10.1007/s13238-015-0182-0 (PMC4491054; doi:10.1007/s13238-015-0182-0)
Supplement: Supplementary file 1 — Supplementary material 1 (DOCX 15 kb) [file 13238_2015_182_MOESM1_ESM.docx]

**Table S1. Data size of each sample used**

| **Samples** | **Number of reads** | **Samples** | **Number of reads** |
| --- | --- | --- | --- |
| **PD1** | **8073004** | **PH1** | **9792960** |
| **PD2** | **11569812** | **PH2** | **10574606** |
| **PD3** | **5293956** | **PH3** | **8892556** |
| **PD4** | **13307752** | **PH4** | **4699594** |
| **PD5** | **18519330** | **PH5** | **7605602** |
| **PD6** | **7600298** | **PH6** | **3270778** |
| **PD7** | **8313926** | **PH7** | **8122434** |
| **PD9** | **12320794** | **PH8** | **11931698** |
| **PD8** | **11841736** | **PH9** | **15200260** |
